# Supplementary material for: The E3 ubiquitin ligase RNF220 maintains hindbrain Hox expression patterns through regulation of WDR5 stability
Source: eLife. 2024 Nov 11;13:RP94657. doi: 10.7554/eLife.94657 (PMC11554307; doi:10.7554/eLife.94657)
Supplement: Supplementary file 1. — Whole-mount brain from E18.5 mice were used (n=2 in WT group and n=3 in Rnf220+/- group). [file elife-94657-supp1.docx]

**Supplementary File 1:** Differently expressed genes identified using microarray between WT and *Rnf220^+/-^* mice.

| **ProbeName** | **WT_4B_NS** | **WT_7B_NS** | **HE_1B_NS** | **HE_2B_NS** | **HE_9B_NS** | **foldchange** | **GeneSymbol** |
| --- | --- | --- | --- | --- | --- | --- | --- |
| A_51_P106443 | 3.9610484 | 3.8312478 | 3.911649 | 3.5465846 | 6.781389 | 0.3269886 | *Ttll4* |
| A_51_P107039 | 5.9857764 | 6.293115 | 7.3528614 | 7.603712 | 8.187723 | 0.3274328 | *4930555G01Rik* |
| A_51_P145453 | 1.1030455 | 1.8843594 | 13.218153 | 13.073017 | 12.635066 | 0.0003574 | *Xist* |
| A_51_P225484 | 3.8190854 | 4.0877843 | 2.6871324 | 1.7105145 | 2.3572092 | 3.146021 | *Epyc* |
| A_51_P232207 | 8.870988 | 6.4653354 | 9.361305 | 9.746093 | 10.04952 | 0.3240584 | *Hoxb6* |
| A_51_P232617 | 1.6583239 | 2.9412262 | 4.149042 | 3.8376906 | 4.293471 | 0.314739 | *Spag17* |
| A_51_P246677 | 10.885349 | 8.35602 | 8.185716 | 7.150078 | 7.7772675 | 5.100983 | *Rec8* |
| A_51_P250590 | 9.816977 | 12.201618 | 9.834278 | 9.617521 | 9.242044 | 3.6539768 | *Sbf2* |
| A_51_P283044 | 7.210637 | 7.1668043 | 1.0895243 | 0.916043 | 1.0501599 | 71.924886 | *Kdm5d* |
| A_51_P303160 | 3.7078912 | 2.96213 | 3.7240596 | 7.054022 | 3.409316 | 0.1996523 | *Arg1* |
| A_51_P305532 | 9.002047 | 9.349363 | 7.1329226 | 7.1292458 | 7.182117 | 4.1062864 | *Eif2s3x* |
| A_51_P307944 | 5.1118865 | 4.8116364 | 1.2979898 | 0.8410812 | 0.8931325 | 15.390296 | *S1pr4* |
| A_51_P353232 | 8.54968 | 5.1697693 | 4.654748 | 5.1941752 | 5.279355 | 6.1218148 | *Tnnc2* |
| A_51_P355122 | 9.005632 | 6.4512677 | 6.390123 | 6.4971147 | 6.698944 | 3.2444464 | *Tnni2* |
| A_51_P398491 | 3.57301 | 3.2548206 | 0.7871613 | 2.121579 | 1.9185144 | 3.2634084 | *Trem1* |
| A_51_P401659 | 8.49288 | 13.804942 | 8.294904 | 8.26872 | 8.293301 | 23.508588 | *Sspn* |
| A_51_P402686 | 5.390537 | 0.7771567 | 6.5473924 | 7.4789324 | 8.404096 | 0.1072456 | *Hoxb8* |
| A_51_P402994 | 7.67957 | 7.889223 | 0.889564 | 0.9211274 | 1.5682712 | 98.803163 | *Ddx3y* |
| A_51_P413147 | 2.9268467 | 3.3636706 | 4.4126472 | 4.0533743 | 5.4956694 | 0.3233795 | *Klk1b3* |
| A_51_P416858 | 7.626576 | 3.8670025 | 4.1174607 | 4.1858187 | 4.177822 | 5.9325753 | *Myl1* |
| A_51_P447189 | 7.250779 | 4.738179 | 5.3955793 | 3.4247038 | 1.976046 | 4.7295439 | *LOC100503215* |
| A_51_P479715 | 8.826565 | 9.364602 | 0.9919275 | 0.9381926 | 1.0508884 | 279.38628 | *Eif2s3y* |
| A_51_P496245 | 3.5971034 | 0.8649594 | 4.750976 | 5.8702064 | 8.07007 | 0.0589677 | *Hoxc6* |
| A_51_P509263 | 5.1390595 | 4.7569666 | 5.2049723 | 6.028042 | 8.534603 | 0.1975087 | *Hoxa7* |
| A_52_P106285 | 7.450177 | 5.0501213 | 4.8360424 | 4.7674747 | 5.396261 | 3.1865628 | *Rnf157* |
| A_52_P113537 | 0.7702979 | 0.7451581 | 9.380402 | 9.468647 | 8.730086 | 0.0028187 | *Xist* |
| A_52_P129547 | 2.2627034 | 2.8756747 | 4.0872307 | 3.994681 | 4.994796 | 0.2808802 | *Cdh10* |
| A_52_P194971 | 7.003126 | 2.4153306 | 7.9176655 | 8.676689 | 9.467014 | 0.1475055 | *Hoxb7* |
| A_52_P245806 | 2.0121722 | 2.2222986 | 8.024733 | 8.075076 | 7.757375 | 0.0174827 | *Tsix* |
| A_52_P247537 | 5.6644816 | 5.820669 | 1.1234794 | 1.0404364 | 1.3392673 | 23.775287 |  |
| A_52_P275069 | 5.1697693 | 5.7891135 | 3.00036 | 3.540889 | 4.4741592 | 3.2708292 | *Gm6792* |
| A_52_P300376 | 0.8213641 | 0.7698782 | 7.833779 | 7.7910423 | 7.6257606 | 0.0080483 | *Xist* |
| A_52_P315942 | 2.6382132 | 1.1713203 | 3.8614466 | 3.9763458 | 3.6737823 | 0.2954844 | *Ptpn6* |
| A_52_P320156 | 1.7486724 | 4.2580724 | 4.245352 | 5.562554 | 5.2181706 | 0.326164 | *E430024C06Rik* |
| A_52_P328825 | 9.731535 | 9.426588 | 11.306289 | 11.323352 | 11.139886 | 0.3138731 | *Wdfy1* |
| A_52_P362360 | 0.9219009 | 0.8588854 | 11.556221 | 11.669298 | 11.221105 | 0.0006426 | *Xist* |
| A_52_P369840 | 4.6016273 | 3.4662702 | 3.311216 | 1.8128297 | 0.9486678 | 3.4481878 | *2900060N12Rik* |
| A_52_P411296 | 9.834575 | 12.287046 | 9.773543 | 9.764503 | 9.759869 | 3.3943711 | *Sh3kbp1* |
| A_52_P415996 | 6.9113474 | 5.664976 | 5.3968697 | 4.112598 | 4.4212303 | 3.1742972 | *Gstm6* |
| A_52_P420563 | 6.4848566 | 12.048418 | 6.416327 | 6.674661 | 6.382933 | 23.939071 | *Ncor2* |
| A_52_P424550 | 1.298509 | 0.9006791 | 4.036758 | 2.8420594 | 3.6169257 | 0.1810222 |  |
| A_52_P459732 | 8.02701 | 6.3407764 | 5.81404 | 5.444841 | 5.9562135 | 3.1675232 | *Rec8* |
| A_52_P620944 | 3.8066573 | 3.6602871 | 1.5122881 | 1.4530756 | 0.9071637 | 5.3516229 |  |
| A_52_P755632 | 3.5135806 | 4.4207397 | 4.49904 | 4.160672 | 8.204504 | 0.1468263 |  |
| A_52_P763332 | 1.06057 | 5.5879946 | 6.3257084 | 7.2547207 | 6.8896036 | 0.2141694 |  |
| A_52_P909122 | 9.128128 | 12.63704 | 10.234246 | 9.10839 | 9.897138 | 3.8354481 |  |
| A_52_P940338 | 0.8494085 | 2.08987 | 4.3664184 | 2.8508143 | 5.45008 | 0.1270092 | *LOC382202* |
| A_52_P980199 | 2.3094199 | 2.2522495 | 4.359635 | 4.6852355 | 3.6413543 | 0.2482597 | *Cdh10* |
| A_52_P98462 | 0.6979232 | 0.7272494 | 2.9216394 | 3.5460274 | 7.518462 | 0.0242659 | *Hoxb9* |
