# Supplementary material for: The E3 ubiquitin ligase RNF220 maintains hindbrain Hox expression patterns through regulation of WDR5 stability
Source: eLife. 2024 Nov 11;13:RP94657. doi: 10.7554/eLife.94657 (PMC11554307; doi:10.7554/eLife.94657)
Supplement: Supplementary file 2. — Whole-mount brain from E18.5 mice were used (n=2 in WT group and n=3 in Rnf220-/- group). [file elife-94657-supp2.docx]

**Supplementary File 2：** Differently expressed genes identified using microarray between WT and *Rnf220^-/-^* mice.

| **ProbeName** | **WT_4B_NS** | **WT_7B_NS** | **KO_3B_NS** | **KO_5B_NS** | **KO_6B_NS** | **foldchange** | **GeneSymbol** |
| --- | --- | --- | --- | --- | --- | --- | --- |
| A_51_P125351 | 6.313856 | 6.601757 | 6.4720597 | 6.4195724 | 10.480064 | 0.1653608 | *Lce1b* |
| A_51_P132185 | 6.5295115 | 6.335476 | 7.368577 | 6.2388043 | 11.309154 | 0.0934783 | *Stfa1* |
| A_51_P135944 | 4.8749924 | 4.083627 | 5.2269435 | 4.498599 | 9.086301 | 0.1150532 | *Stfa2* |
| A_51_P170059 | 3.6045246 | 4.311549 | 3.71578 | 3.9532177 | 10.690251 | 0.0285741 | *Lce1m* |
| A_51_P183197 | 4.167489 | 4.6221843 | 4.5278687 | 4.208487 | 8.697207 | 0.1399295 | *2310002J15Rik* |
| A_51_P214127 | 3.6033967 | 2.9677386 | 3.4971817 | 4.097985 | 8.025378 | 0.1037036 | *Cpa3* |
| A_51_P232207 | 8.870988 | 6.4653354 | 9.991578 | 10.203091 | 9.469767 | 0.2872938 | *Hoxb6* |
| A_51_P250590 | 9.816977 | 12.201618 | 9.5322485 | 9.307759 | 9.114188 | 4.3656323 | *Sbf2* |
| A_51_P260265 | 6.1705537 | 5.226247 | 7.260891 | 7.727149 | 7.3651867 | 0.3097302 | *Hoxd4* |
| A_51_P287198 | 4.01975 | 4.0344496 | 3.9391453 | 3.7721603 | 7.729372 | 0.2027683 | *Krt23* |
| A_51_P296109 | 4.4744844 | 3.92833 | 4.659961 | 4.587028 | 7.9215555 | 0.1925611 | *Dmkn* |
| A_51_P303784 | 4.194939 | 3.565834 | 1.1152956 | 1.1867241 | 2.18678 | 5.0285685 | *Olfr218* |
| A_51_P307944 | 5.1118865 | 4.8116364 | 0.8316174 | 0.7184657 | 0.8546457 | 17.960621 | *S1pr4* |
| A_51_P321011 | 6.994348 | 7.199608 | 3.8395464 | 4.032716 | 3.4444318 | 9.9049398 |  |
| A_51_P336830 | 4.502313 | 2.8254704 | 3.985986 | 3.6439693 | 8.376842 | 0.1237058 | *Fabp4* |
| A_51_P336833 | 4.593224 | 3.1738102 | 4.121704 | 3.8008437 | 8.382238 | 0.1362804 | *Fabp4* |
| A_51_P384894 | 7.477057 | 7.303433 | 8.060354 | 7.1584826 | 12.393815 | 0.0870583 | *Csta* |
| A_51_P393968 | 5.735548 | 5.217275 | 2.2287815 | 1.0561386 | 1.0544479 | 15.346941 | *Vsx2* |
| A_51_P401659 | 8.49288 | 13.804942 | 8.375488 | 8.372072 | 8.301443 | 22.48262 | *Sspn* |
| A_51_P402686 | 5.390537 | 0.7771567 | 8.055451 | 8.566343 | 6.7811 | 0.0867356 | *Hoxb8* |
| A_51_P444543 | 5.1029267 | 5.1083794 | 3.17601 | 2.371889 | 2.719416 | 4.9660082 | *Dbh* |
| A_51_P461040 | 6.6832285 | 6.5802245 | 6.843542 | 6.9395475 | 10.69976 | 0.1566059 | *Crct1* |
| A_51_P474752 | 3.7151783 | 3.267671 | 0.8849869 | 0.8739997 | 0.8531357 | 6.2244189 | *Ucn2* |
| A_51_P496245 | 3.5971034 | 0.8649594 | 6.764801 | 8.603234 | 4.844242 | 0.0396761 | *Hoxc6* |
| A_51_P499698 | 5.7629433 | 5.356135 | 5.2179017 | 5.541067 | 10.220467 | 0.1119105 | *Asprv1* |
| A_51_P504815 | 5.939404 | 5.932974 | 6.521724 | 5.831172 | 12.178275 | 0.0384009 | *Stfa3* |
| A_51_P509263 | 5.1390595 | 4.7569666 | 7.245264 | 8.561127 | 5.753749 | 0.1601265 | *Hoxa7* |
| A_51_P514035 | 5.6431694 | 4.7971907 | 5.6459165 | 6.3477407 | 10.138423 | 0.0926953 | *Cma1* |
| A_52_P117920 | 5.120014 | 3.3515055 | 3.6814542 | 3.3344727 | 8.7837105 | 0.1455336 |  |
| A_52_P117922 | 4.337855 | 4.06896 | 4.056633 | 3.9852946 | 9.184422 | 0.0903605 |  |
| A_52_P171692 | 5.2034197 | 4.5558033 | 5.1785574 | 4.851259 | 8.4255705 | 0.2214345 | *1190003J15Rik* |
| A_52_P194971 | 7.003126 | 2.4153306 | 9.013775 | 9.578628 | 8.266773 | 0.1260852 | *Hoxb7* |
| A_52_P240842 | 4.914063 | 1.2371906 | 5.9930615 | 6.191735 | 5.1821957 | 0.2816936 | *9430077A04Rik* |
| A_52_P347956 | 0.7941967 | 0.9586504 | 4.051279 | 4.083244 | 3.9578302 | 0.1124228 | *Cyp2j13* |
| A_52_P411296 | 9.834575 | 12.287046 | 9.918812 | 9.747645 | 9.758842 | 3.2910543 | *Sh3kbp1* |
| A_52_P420563 | 6.4848566 | 12.048418 | 6.4241385 | 6.346026 | 6.5670357 | 24.760186 | *Ncor2* |
| A_52_P436122 | 2.9213138 | 3.0083542 | 5.30703 | 5.467043 | 5.6950293 | 0.1727731 |  |
| A_52_P445239 | 3.697067 | 5.3639936 | 3.4139938 | 3.297052 | 2.1909828 | 3.2421623 | *Plp1* |
| A_52_P487686 | 6.869822 | 6.442713 | 7.520336 | 6.6244674 | 11.930332 | 0.0730948 | *BC100530* |
| A_52_P523368 | 4.5968947 | 4.545647 | 4.597747 | 4.583084 | 7.504041 | 0.3105278 | *Psapl1* |
| A_52_P531325 | 5.7284603 | 5.8114233 | 1.0499647 | 1.0681556 | 2.071632 | 19.56396 | *Rps6* |
| A_52_P620944 | 3.8066573 | 3.6602871 | 0.8701608 | 0.7867022 | 0.8770287 | 7.4133046 |  |
| A_52_P673122 | 3.6899672 | 3.9134355 | 2.7354527 | 0.9638019 | 2.2010896 | 3.1768653 |  |
| A_52_P909122 | 9.128128 | 12.63704 | 10.752602 | 9.08155 | 9.953282 | 3.1899222 |  |
| A_52_P98614 | 6.431283 | 5.7046127 | 1.0239266 | 1.010514 | 0.9768893 | 34.518845 | *Slc6a2* |
