# Supplementary material for: The E3 ubiquitin ligase RNF220 maintains hindbrain Hox expression patterns through regulation of WDR5 stability
Source: eLife. 2024 Nov 11;13:RP94657. doi: 10.7554/eLife.94657 (PMC11554307; doi:10.7554/eLife.94657)
Supplement: Supplementary file 3. — The pons from 2 months’ mice were used (n=3 mice per group). [file elife-94657-supp3.docx]

**Supplementary File 3:** Uniquely and highly expressed genes of each cluster in snRNA-seq.

| **Genes** | **p_val** | **avg_logFC** | **pct.1** | **pct.2** | **cluster** |
| --- | --- | --- | --- | --- | --- |
| *Snhg11* | 0 | 0.9540666 | 0.993 | 0.814 | 0 |
| *Zfp101* | 0 | 0.7367873 | 0.832 | 0.746 | 0 |
| *Ube3a* | 0 | 0.5335392 | 0.613 | 0.483 | 0 |
| *D10Wsu102e* | 0 | 0.4966353 | 0.707 | 0.571 | 0 |
| *Srrm2* | 0 | 0.4596324 | 0.663 | 0.549 | 0 |
| *AC149090.1* | 0 | 0.4474371 | 0.441 | 0.301 | 0 |
| *Mmachc* | 0 | 0.4081537 | 0.411 | 0.296 | 0 |
| *Vmn2r53* | 0 | 0.375206 | 0.426 | 0.302 | 0 |
| *Pnisr* | 0 | 0.3638224 | 0.487 | 0.389 | 0 |
| *Rbm25* | 0 | 0.3391598 | 0.515 | 0.454 | 0 |
| *Nktr* | 0 | 0.3348808 | 0.401 | 0.305 | 0 |
| *Fus* | 0 | 0.3149181 | 0.424 | 0.352 | 0 |
| *Syne1* | 0 | 0.3130419 | 0.5 | 0.447 | 0 |
| *Gm17132* | 0 | 0.3126096 | 0.349 | 0.257 | 0 |
| *Usp19* | 0 | 0.2997827 | 0.335 | 0.265 | 0 |
| *Cenpc1* | 0 | 0.2940808 | 0.284 | 0.199 | 0 |
| *Gm48119* | 0 | 0.2927253 | 0.306 | 0.21 | 0 |
| *Vmn2r76* | 0 | 0.2875166 | 0.301 | 0.208 | 0 |
| *Gria2* | 0 | 0.2844672 | 0.458 | 0.404 | 0 |
| *Luc7l2* | 0 | 0.2652363 | 0.33 | 0.271 | 0 |
| *Dennd1b* | 0 | 0.2634844 | 0.282 | 0.212 | 0 |
| *Tmem72* | 0 | 0.257478 | 0.34 | 0.287 | 0 |
| *Epha5* | 0 | 0.2546523 | 0.332 | 0.291 | 0 |
| *Snrnp70* | 0 | 0.2524077 | 0.35 | 0.296 | 0 |
| *Nap1l5* | 0 | 0.6473373 | 0.758 | 0.393 | 1 |
| *Gad2* | 0 | 0.4701617 | 0.375 | 0.121 | 1 |
| *Scg2* | 0 | 0.4498357 | 0.503 | 0.249 | 1 |
| *Ahi1* | 0 | 0.4384739 | 0.506 | 0.197 | 1 |
| *Gprasp1* | 0 | 0.3767198 | 0.609 | 0.364 | 1 |
| *6330403K07Rik* | 0 | 0.3761973 | 0.358 | 0.077 | 1 |
| *Tmem130* | 0 | 0.3663431 | 0.466 | 0.22 | 1 |
| *Celf4* | 0 | 0.3487743 | 0.501 | 0.241 | 1 |
| *Peg3* | 0 | 0.3147951 | 0.775 | 0.588 | 1 |
| *Impact* | 0 | 0.3073325 | 0.433 | 0.22 | 1 |
| *Gap43* | 0 | 0.2998809 | 0.325 | 0.107 | 1 |
| *Pnmal2* | 0 | 0.297587 | 0.433 | 0.22 | 1 |
| *Zwint* | 0 | 0.2867501 | 0.526 | 0.333 | 1 |
| *Resp18* | 0 | 0.2630204 | 0.322 | 0.167 | 1 |
| *Nefm* | 0 | 0.7882714 | 0.875 | 0.396 | 2 |
| *Snap25* | 0 | 0.6622256 | 0.93 | 0.556 | 2 |
| *Nefh* | 0 | 0.6536582 | 0.736 | 0.249 | 2 |
| *Map1b* | 0 | 0.6242659 | 0.958 | 0.646 | 2 |
| *Atp1b1* | 0 | 0.6146502 | 0.954 | 0.669 | 2 |
| *Cplx1* | 0 | 0.6064388 | 0.795 | 0.319 | 2 |
| *Sparcl1* | 0 | 0.5704161 | 0.825 | 0.366 | 2 |
| *Calm1* | 0 | 0.5449357 | 0.902 | 0.611 | 2 |
| *Atp1a3* | 0 | 0.5418078 | 0.765 | 0.448 | 2 |
| *Nefl* | 0 | 0.5120509 | 0.704 | 0.297 | 2 |
| *Pkm* | 0 | 0.4929712 | 0.82 | 0.423 | 2 |
| *Vsnl1* | 0 | 0.4829055 | 0.733 | 0.305 | 2 |
| *Map1a* | 0 | 0.4735071 | 0.773 | 0.383 | 2 |
| *Lars2* | 0 | 0.4709724 | 0.969 | 0.834 | 2 |
| *Syt2* | 0 | 0.4691961 | 0.684 | 0.247 | 2 |
| *Kcnc3* | 0 | 0.4638185 | 0.599 | 0.199 | 2 |
| *Slc25a4* | 0 | 0.4524251 | 0.791 | 0.461 | 2 |
| *Kcna2* | 0 | 0.4155329 | 0.699 | 0.288 | 2 |
| *Vamp1* | 0 | 0.4013327 | 0.687 | 0.27 | 2 |
| *Lamp5* | 0 | 0.3926798 | 0.501 | 0.15 | 2 |
| *Slc6a5* | 0 | 0.3915305 | 0.373 | 0.108 | 2 |
| *Ndrg4* | 0 | 0.3745433 | 0.75 | 0.378 | 2 |
| *Ckb* | 0 | 0.3706504 | 0.725 | 0.374 | 2 |
| *Map7d2* | 0 | 0.3696816 | 0.65 | 0.294 | 2 |
| *Hsp90aa1* | 0 | 0.3623147 | 0.815 | 0.496 | 2 |
| *Stmn3* | 0 | 0.36053 | 0.67 | 0.334 | 2 |
| *Kcna1* | 0 | 0.3599023 | 0.601 | 0.222 | 2 |
| *Gm20594* | 0 | 0.3511162 | 0.862 | 0.561 | 2 |
| *Mdh1* | 0 | 0.3501233 | 0.685 | 0.325 | 2 |
| *Calm3* | 0 | 0.3483847 | 0.663 | 0.301 | 2 |
| *Gapdh* | 0 | 0.3429265 | 0.598 | 0.371 | 2 |
| *Calm2* | 0 | 0.342898 | 0.8 | 0.465 | 2 |
| *Ldhb* | 0 | 0.3426175 | 0.638 | 0.264 | 2 |
| *Ghitm* | 0 | 0.3340682 | 0.647 | 0.273 | 2 |
| *Ina* | 0 | 0.3311065 | 0.563 | 0.21 | 2 |
| *Atp5b* | 0 | 0.3296494 | 0.76 | 0.429 | 2 |
| *Ywhag* | 0 | 0.3289253 | 0.674 | 0.305 | 2 |
| *Hspa8* | 0 | 0.3254792 | 0.695 | 0.394 | 2 |
| *Scn1a* | 0 | 0.3190687 | 0.688 | 0.33 | 2 |
| *mt-Nd1* | 0 | 0.3189891 | 0.65 | 0.327 | 2 |
| *mt-Cytb* | 0 | 0.3017935 | 0.723 | 0.395 | 2 |
| *Napb* | 0 | 0.2963015 | 0.71 | 0.386 | 2 |
| *Nars* | 0 | 0.295612 | 0.617 | 0.287 | 2 |
| *Hsp90ab1* | 0 | 0.294607 | 0.843 | 0.561 | 2 |
| *Nat8l* | 0 | 0.2926316 | 0.591 | 0.204 | 2 |
| *Rtn3* | 0 | 0.2911262 | 0.705 | 0.371 | 2 |
| *Aldoc* | 0 | 0.2879719 | 0.629 | 0.286 | 2 |
| *Atp2b2* | 0 | 0.282642 | 0.651 | 0.311 | 2 |
| *Dnm1* | 0 | 0.2807882 | 0.604 | 0.289 | 2 |
| *Eno2* | 0 | 0.2807822 | 0.647 | 0.297 | 2 |
| *Glra1* | 0 | 0.2801859 | 0.558 | 0.213 | 2 |
| *Sv2c* | 0 | 0.2795559 | 0.472 | 0.151 | 2 |
| *Scn8a* | 0 | 0.277919 | 0.621 | 0.277 | 2 |
| *Rph3a* | 0 | 0.2774697 | 0.519 | 0.173 | 2 |
| *Atp2a2* | 0 | 0.2686874 | 0.642 | 0.309 | 2 |
| *Nsf* | 0 | 0.2676745 | 0.696 | 0.369 | 2 |
| *Slc24a2* | 0 | 0.26494 | 0.594 | 0.254 | 2 |
| *Atp6v1a* | 0 | 0.2648543 | 0.658 | 0.341 | 2 |
| *Cltc* | 0 | 0.2623385 | 0.617 | 0.293 | 2 |
| *mt-Nd2* | 0 | 0.252835 | 0.532 | 0.269 | 2 |
| *Clstn1* | 0 | 0.250687 | 0.632 | 0.3 | 2 |
| *Plp1* | 0 | 3.4437013 | 0.991 | 0.211 | 3 |
| *Trf* | 0 | 1.8707847 | 0.803 | 0.058 | 3 |
| *Mag* | 0 | 1.7196174 | 0.799 | 0.033 | 3 |
| *Apod* | 0 | 1.6560469 | 0.607 | 0.039 | 3 |
| *Fth1* | 0 | 1.6308212 | 0.792 | 0.436 | 3 |
| *Ptgds* | 0 | 1.5264231 | 0.343 | 0.098 | 3 |
| *Scd2* | 0 | 1.4443092 | 0.764 | 0.222 | 3 |
| *Mbp* | 0 | 1.4301111 | 0.782 | 0.14 | 3 |
| *Cnp* | 0 | 1.4203155 | 0.663 | 0.036 | 3 |
| *Mobp* | 0 | 1.3381085 | 0.696 | 0.087 | 3 |
| *Car2* | 0 | 1.2150987 | 0.543 | 0.046 | 3 |
| *Mal* | 0 | 1.2013232 | 0.523 | 0.02 | 3 |
| *Qk* | 0 | 1.1079033 | 0.594 | 0.064 | 3 |
| *Gpm6b* | 0 | 1.0702732 | 0.564 | 0.071 | 3 |
| *Aplp1* | 0 | 1.0540449 | 0.636 | 0.222 | 3 |
| *Ermn* | 0 | 0.987124 | 0.471 | 0.012 | 3 |
| *Glul* | 0 | 0.9430473 | 0.453 | 0.064 | 3 |
| *Slc12a2* | 0 | 0.8725496 | 0.479 | 0.075 | 3 |
| *Ugt8a* | 0 | 0.8402525 | 0.427 | 0.038 | 3 |
| *Ptprd* | 0 | 0.820107 | 0.602 | 0.295 | 3 |
| *Enpp2* | 0 | 0.7993549 | 0.648 | 0.088 | 3 |
| *S100b* | 0 | 0.7940206 | 0.373 | 0.13 | 3 |
| *Tubb4a* | 0 | 0.7869016 | 0.431 | 0.212 | 3 |
| *Mog* | 0 | 0.7455389 | 0.362 | 0.009 | 3 |
| *Cryab* | 0 | 0.6964547 | 0.316 | 0.018 | 3 |
| *Gjc3* | 0 | 0.6954245 | 0.353 | 0.023 | 3 |
| *Abca2* | 0 | 0.6672388 | 0.462 | 0.246 | 3 |
| *Cntn2* | 0 | 0.6642329 | 0.376 | 0.115 | 3 |
| *Cldn11* | 0 | 0.6627474 | 0.303 | 0.008 | 3 |
| *Zeb2* | 0 | 0.6306229 | 0.347 | 0.047 | 3 |
| *Stmn4* | 0 | 0.6265405 | 0.339 | 0.059 | 3 |
| *Map7* | 0 | 0.6238302 | 0.331 | 0.042 | 3 |
| *Gsn* | 0 | 0.602966 | 0.279 | 0.007 | 3 |
| *Ptma* | 0 | 0.5983608 | 0.353 | 0.133 | 3 |
| *Klk6* | 0 | 0.596435 | 0.236 | 0.003 | 3 |
| *Syt11* | 0 | 0.5895886 | 0.669 | 0.548 | 3 |
| *Fnbp1* | 0 | 0.5812498 | 0.383 | 0.168 | 3 |
| *Actb* | 0 | 0.5724746 | 0.628 | 0.52 | 3 |
| *Qdpr* | 0 | 0.5626731 | 0.286 | 0.072 | 3 |
| *Cmtm5* | 0 | 0.5616848 | 0.263 | 0.009 | 3 |
| *Kcnj10* | 0 | 0.5520853 | 0.288 | 0.024 | 3 |
| *Lamp1* | 0 | 0.531557 | 0.335 | 0.153 | 3 |
| *Hapln2* | 0 | 0.5287375 | 0.247 | 0.006 | 3 |
| *Pex5l* | 0 | 0.5259814 | 0.304 | 0.091 | 3 |
| *Ndrg1* | 0 | 0.5249046 | 0.256 | 0.012 | 3 |
| *Tmem88b* | 0 | 0.5231319 | 0.254 | 0.022 | 3 |
| *Efcab14* | 0 | 0.5167435 | 0.279 | 0.077 | 3 |
| *Dock9* | 0 | 0.5114567 | 0.318 | 0.137 | 3 |
| *Kif1b* | 0 | 0.5100938 | 0.607 | 0.504 | 3 |
| *Tmem151a* | 0 | 0.5016586 | 0.289 | 0.116 | 3 |
| *Prr5l* | 0 | 0.4861403 | 0.23 | 0.007 | 3 |
| *Plxnb3* | 0 | 0.4751939 | 0.211 | 0.007 | 3 |
| *Rtn4* | 0 | 0.4746112 | 0.425 | 0.323 | 3 |
| *Tspan2* | 0 | 0.4740951 | 0.218 | 0.017 | 3 |
| *Adamts4* | 0 | 0.4727044 | 0.231 | 0.009 | 3 |
| *Dock10* | 0 | 0.4721793 | 0.255 | 0.042 | 3 |
| *Cd81* | 0 | 0.4688141 | 0.309 | 0.174 | 3 |
| *Sez6l2* | 0 | 0.4638343 | 0.326 | 0.206 | 3 |
| *Selenop* | 0 | 0.4599166 | 0.215 | 0.018 | 3 |
| *Aatk* | 0 | 0.4584498 | 0.264 | 0.106 | 3 |
| *Hmgcs1* | 0 | 0.4515978 | 0.299 | 0.18 | 3 |
| *Pcdh9* | 0 | 0.4486376 | 0.445 | 0.323 | 3 |
| *Phldb1* | 0 | 0.4436813 | 0.204 | 0.017 | 3 |
| *Tuba1a* | 0 | 0.4378003 | 0.347 | 0.26 | 3 |
| *Tmem63a* | 0 | 0.4376406 | 0.207 | 0.005 | 3 |
| *Kndc1* | 0 | 0.4354788 | 0.289 | 0.165 | 3 |
| *Slc48a1* | 0 | 0.4345389 | 0.241 | 0.071 | 3 |
| *Plekhh1* | 0 | 0.4289592 | 0.2 | 0.015 | 3 |
| *Ywhaq* | 0 | 0.4219728 | 0.318 | 0.223 | 3 |
| *Edil3* | 0 | 0.4199897 | 0.285 | 0.145 | 3 |
| *Fgfr2* | 0 | 0.4143634 | 0.2 | 0.02 | 3 |
| *Otud7b* | 0 | 0.4113574 | 0.231 | 0.059 | 3 |
| *Efnb3* | 0 | 0.4088633 | 0.202 | 0.009 | 3 |
| *Anln* | 0 | 0.400709 | 0.194 | 0.015 | 3 |
| *Rnf13* | 0 | 0.3953748 | 0.238 | 0.088 | 3 |
| *Agpat4* | 0 | 0.3866612 | 0.207 | 0.036 | 3 |
| *Cdc37l1* | 0 | 0.3860095 | 0.285 | 0.171 | 3 |
| *Gjc2* | 0 | 0.3806491 | 0.179 | 0.004 | 3 |
| *Lpar1* | 0 | 0.3783473 | 0.185 | 0.007 | 3 |
| *Adipor2* | 0 | 0.3777323 | 0.187 | 0.035 | 3 |
| *Secisbp2l* | 0 | 0.3754237 | 0.212 | 0.06 | 3 |
| *Fez1* | 0 | 0.3746373 | 0.249 | 0.142 | 3 |
| *Myrf* | 0 | 0.3745537 | 0.185 | 0.021 | 3 |
| *Nfasc* | 0 | 0.371303 | 0.303 | 0.265 | 3 |
| *Pmp22* | 0 | 0.3701979 | 0.164 | 0.012 | 3 |
| *Sirt2* | 0 | 0.3635241 | 0.21 | 0.071 | 3 |
| *Shisa4* | 0 | 0.3625061 | 0.215 | 0.09 | 3 |
| *Tmeff2* | 0 | 0.3618407 | 0.214 | 0.075 | 3 |
| *Kif13b* | 0 | 0.3591705 | 0.195 | 0.047 | 3 |
| *Sec11c* | 0 | 0.3536522 | 0.207 | 0.079 | 3 |
| *Daam2* | 0 | 0.3526074 | 0.182 | 0.037 | 3 |
| *Slc44a1* | 0 | 0.3515341 | 0.189 | 0.04 | 3 |
| *Arhgap23* | 0 | 0.3510998 | 0.18 | 0.04 | 3 |
| *Itm2b* | 0 | 0.3506868 | 0.356 | 0.302 | 3 |
| *Cpd* | 0 | 0.3491617 | 0.188 | 0.052 | 3 |
| *Zdhhc20* | 0 | 0.3481961 | 0.257 | 0.161 | 3 |
| *Pacs2* | 0 | 0.347316 | 0.198 | 0.063 | 3 |
| *Wscd1* | 0 | 0.341744 | 0.175 | 0.037 | 3 |
| *Tppp3* | 0 | 0.336165 | 0.191 | 0.088 | 3 |
| *Ttyh2* | 0 | 0.3342392 | 0.159 | 0.008 | 3 |
| *Atp1b3* | 0 | 0.329504 | 0.189 | 0.07 | 3 |
| *Aspa* | 0 | 0.3264428 | 0.158 | 0.014 | 3 |
| *Stmn1* | 0 | 0.3257563 | 0.228 | 0.123 | 3 |
| *Pea15a* | 0 | 0.3252495 | 0.203 | 0.09 | 3 |
| *Etv1* | 0 | 0.32521 | 0.152 | 0.014 | 3 |
| *Plin3* | 0 | 0.3230782 | 0.148 | 0.01 | 3 |
| *Gjb1* | 0 | 0.3226736 | 0.148 | 0.002 | 3 |
| *Taldo1* | 0 | 0.322439 | 0.168 | 0.04 | 3 |
| *Serpinb1a* | 0 | 0.3173902 | 0.143 | 0.003 | 3 |
| *Arhgef10* | 0 | 0.3167752 | 0.151 | 0.013 | 3 |
| *Usp54* | 0 | 0.3166988 | 0.171 | 0.044 | 3 |
| *Dbndd2* | 0 | 0.3153281 | 0.172 | 0.05 | 3 |
| *Enpp4* | 0 | 0.3150774 | 0.162 | 0.035 | 3 |
| *Elovl1* | 0 | 0.3143994 | 0.15 | 0.009 | 3 |
| *Sec14l5* | 0 | 0.314212 | 0.147 | 0.014 | 3 |
| *Wnk1* | 0 | 0.3140146 | 0.257 | 0.188 | 3 |
| *Magt1* | 0 | 0.3065059 | 0.155 | 0.026 | 3 |
| *Npc1* | 0 | 0.3014844 | 0.184 | 0.074 | 3 |
| *Psat1* | 0 | 0.3003193 | 0.147 | 0.022 | 3 |
| *Cpox* | 0 | 0.299684 | 0.161 | 0.048 | 3 |
| *Slc38a2* | 0 | 0.2976823 | 0.226 | 0.164 | 3 |
| *Tppp* | 0 | 0.2973396 | 0.181 | 0.086 | 3 |
| *Ptn* | 0 | 0.2962332 | 0.179 | 0.099 | 3 |
| *Pik3c2b* | 0 | 0.2950909 | 0.164 | 0.047 | 3 |
| *Gltp* | 0 | 0.2946176 | 0.14 | 0.009 | 3 |
| *Kctd3* | 0 | 0.2930424 | 0.206 | 0.115 | 3 |
| *Cers2* | 0 | 0.2929419 | 0.151 | 0.018 | 3 |
| *Kctd13* | 0 | 0.2920035 | 0.146 | 0.025 | 3 |
| *Phlpp1* | 0 | 0.291286 | 0.154 | 0.031 | 3 |
| *Eef1a1* | 0 | 0.2910657 | 0.259 | 0.19 | 3 |
| *Mast4* | 0 | 0.2900613 | 0.213 | 0.133 | 3 |
| *Myo6* | 0 | 0.2893583 | 0.175 | 0.061 | 3 |
| *Ankrd28* | 0 | 0.2875955 | 0.175 | 0.066 | 3 |
| *Fa2h* | 0 | 0.2862184 | 0.135 | 0.005 | 3 |
| *Sort1* | 0 | 0.284055 | 0.194 | 0.121 | 3 |
| *Nrbp2* | 0 | 0.2810097 | 0.158 | 0.047 | 3 |
| *Bin1* | 0 | 0.2803092 | 0.254 | 0.219 | 3 |
| *Eml1* | 0 | 0.2798412 | 0.145 | 0.022 | 3 |
| *Ppp1r16b* | 0 | 0.2772155 | 0.162 | 0.061 | 3 |
| *Csrp1* | 0 | 0.2769179 | 0.129 | 0.013 | 3 |
| *Plekhb1* | 0 | 0.2768113 | 0.136 | 0.012 | 3 |
| *Olig1* | 0 | 0.2754197 | 0.127 | 0.006 | 3 |
| *Foxn3* | 0 | 0.2744346 | 0.185 | 0.107 | 3 |
| *Sccpdh* | 0 | 0.2725627 | 0.164 | 0.073 | 3 |
| *Dpy19l1* | 0 | 0.2709104 | 0.146 | 0.036 | 3 |
| *Gnao1* | 0 | 0.2688801 | 0.284 | 0.253 | 3 |
| *Elovl7* | 0 | 0.2688145 | 0.134 | 0.019 | 3 |
| *Sypl* | 0 | 0.2686733 | 0.147 | 0.034 | 3 |
| *Inf2* | 0 | 0.2684947 | 0.129 | 0.008 | 3 |
| *S100a16* | 0 | 0.2631298 | 0.119 | 0.005 | 3 |
| *Arrdc3* | 0 | 0.2628438 | 0.135 | 0.028 | 3 |
| *Tmbim1* | 0 | 0.2586548 | 0.157 | 0.054 | 3 |
| *Arhgef2* | 0 | 0.2572418 | 0.151 | 0.047 | 3 |
| *Car14* | 0 | 0.2563609 | 0.126 | 0.008 | 3 |
| *Josd2* | 0 | 0.2548116 | 0.135 | 0.028 | 3 |
| *Arsg* | 0 | 0.2546638 | 0.122 | 0.013 | 3 |
| *Ccp110* | 0 | 0.2527608 | 0.139 | 0.05 | 3 |
| *Ckb1* | 0 | 0.4836905 | 0.724 | 0.433 | 4 |
| *Pvalb* | 0 | 0.4661873 | 0.413 | 0.109 | 4 |
| *Sphkap* | 0 | 0.438224 | 0.399 | 0.08 | 4 |
| *Ppp3ca* | 0 | 0.4227978 | 0.525 | 0.304 | 4 |
| *Sparcl11* | 0 | 0.4128678 | 0.744 | 0.447 | 4 |
| *Necab2* | 0 | 0.3931824 | 0.31 | 0.025 | 4 |
| *Scn4b* | 0 | 0.3793392 | 0.361 | 0.137 | 4 |
| *Map1a1* | 0 | 0.3768442 | 0.704 | 0.452 | 4 |
| *Nefm1* | 0 | 0.3686965 | 0.809 | 0.479 | 4 |
| *Nefl1* | 0 | 0.3585811 | 0.663 | 0.367 | 4 |
| *Glra11* | 0 | 0.3544084 | 0.505 | 0.273 | 4 |
| *Slc17a6* | 0 | 0.3484613 | 0.504 | 0.209 | 4 |
| *AI593442* | 0 | 0.3483056 | 0.371 | 0.138 | 4 |
| *Vsnl11* | 0 | 0.343443 | 0.618 | 0.381 | 4 |
| *Snap251* | 0 | 0.341713 | 0.862 | 0.622 | 4 |
| *Atp1b11* | 0 | 0.3404215 | 0.922 | 0.718 | 4 |
| *Cplx11* | 0 | 0.3299008 | 0.644 | 0.405 | 4 |
| *Atp5b1* | 0 | 0.3276665 | 0.695 | 0.487 | 4 |
| *Ebf1* | 0 | 0.3085761 | 0.297 | 0.08 | 4 |
| *Arpp21* | 0 | 0.304836 | 0.389 | 0.139 | 4 |
| *Calm21* | 0 | 0.3026335 | 0.723 | 0.524 | 4 |
| *Ntng1* | 0 | 0.3000027 | 0.285 | 0.063 | 4 |
| *Sptb* | 0 | 0.2974729 | 0.308 | 0.133 | 4 |
| *Tanc1* | 0 | 0.2920494 | 0.295 | 0.078 | 4 |
| *Cplx2* | 0 | 0.2859935 | 0.31 | 0.131 | 4 |
| *Chgb* | 0 | 0.2855212 | 0.485 | 0.303 | 4 |
| *Ldhb1* | 0 | 0.2806631 | 0.53 | 0.331 | 4 |
| *Vamp11* | 0 | 0.2794603 | 0.546 | 0.346 | 4 |
| *Rgs7bp* | 0 | 0.2751283 | 0.381 | 0.198 | 4 |
| *Pitpnc1* | 0 | 0.2680408 | 0.283 | 0.094 | 4 |
| *Ina1* | 0 | 0.2599847 | 0.441 | 0.274 | 4 |
| *Plcb4* | 0 | 0.2589942 | 0.389 | 0.219 | 4 |
| *Smarca2* | 0 | 0.2534591 | 0.459 | 0.295 | 4 |
| *Stmn31* | 1.78E-266 | 0.2545976 | 0.552 | 0.396 | 4 |
| *Atp1a31* | 6.78E-265 | 0.3353992 | 0.668 | 0.505 | 4 |
| *Apoe* | 0 | 1.6086973 | 0.577 | 0.055 | 5 |
| *Atp1a2* | 0 | 1.5247581 | 0.674 | 0.031 | 5 |
| *Sparc* | 0 | 1.0838668 | 0.476 | 0.03 | 5 |
| *Slc1a2* | 0 | 1.0801745 | 0.466 | 0.047 | 5 |
| *Ptgds1* | 0 | 1.0527407 | 0.236 | 0.113 | 5 |
| *Cst3* | 0 | 0.9195035 | 0.414 | 0.152 | 5 |
| *Atp1b2* | 0 | 0.7810754 | 0.351 | 0.044 | 5 |
| *Aqp4* | 0 | 0.6686322 | 0.258 | 0.005 | 5 |
| *Slc4a4* | 0 | 0.6680274 | 0.297 | 0.028 | 5 |
| *Rgs5* | 0 | 0.6562534 | 0.187 | 0.007 | 5 |
| *Gm205941* | 0 | 0.6442261 | 0.675 | 0.621 | 5 |
| *Mbp1* | 0 | 0.6325801 | 0.391 | 0.181 | 5 |
| *Sparcl12* | 0 | 0.631754 | 0.618 | 0.454 | 5 |
| *Ndrg2* | 0 | 0.6043657 | 0.3 | 0.057 | 5 |
| *Flt1* | 0 | 0.5983766 | 0.194 | 0.007 | 5 |
| *Gpr37l1* | 0 | 0.589757 | 0.257 | 0.008 | 5 |
| *Slc6a11* | 0 | 0.583096 | 0.247 | 0.009 | 5 |
| *Aldoc1* | 0 | 0.5646454 | 0.446 | 0.353 | 5 |
| *Bcan* | 0 | 0.5470201 | 0.265 | 0.121 | 5 |
| *Gja1* | 0 | 0.5269379 | 0.231 | 0.01 | 5 |
| *Bsg* | 0 | 0.5178038 | 0.325 | 0.315 | 5 |
| *Cd811* | 0 | 0.4869174 | 0.331 | 0.179 | 5 |
| *Ttyh1* | 0 | 0.4798814 | 0.311 | 0.149 | 5 |
| *Fgfr3* | 0 | 0.4471816 | 0.187 | 0.003 | 5 |
| *Vtn* | 0 | 0.4422136 | 0.121 | 0.002 | 5 |
| *Mobp1* | 0 | 0.4404429 | 0.297 | 0.127 | 5 |
| *Ly6c1* | 0 | 0.4356071 | 0.124 | 0.002 | 5 |
| *Pla2g7* | 0 | 0.4336423 | 0.215 | 0.067 | 5 |
| *Htra1* | 0 | 0.4315661 | 0.179 | 0.03 | 5 |
| *Zbtb20* | 0 | 0.4251622 | 0.461 | 0.352 | 5 |
| *Mt1* | 0 | 0.4185149 | 0.184 | 0.03 | 5 |
| *Kcnj101* | 0 | 0.4136619 | 0.217 | 0.038 | 5 |
| *Slc7a10* | 0 | 0.4054401 | 0.161 | 0.006 | 5 |
| *Clu* | 0 | 0.3963422 | 0.271 | 0.229 | 5 |
| *Itih3* | 0 | 0.3954393 | 0.149 | 0.004 | 5 |
| *Glul1* | 0 | 0.3948758 | 0.261 | 0.088 | 5 |
| *Macf1* | 0 | 0.389849 | 0.47 | 0.415 | 5 |
| *Scd21* | 0 | 0.3857611 | 0.387 | 0.258 | 5 |
| *Qk1* | 0 | 0.3819904 | 0.29 | 0.098 | 5 |
| *Pbxip1* | 0 | 0.3818055 | 0.18 | 0.021 | 5 |
| *mt-Cytb1* | 0 | 0.3775106 | 0.488 | 0.461 | 5 |
| *Ntsr2* | 0 | 0.3774527 | 0.153 | 0.004 | 5 |
| *Son* | 0 | 0.3771165 | 0.412 | 0.339 | 5 |
| *Fth11* | 0 | 0.3770047 | 0.508 | 0.461 | 5 |
| *Ptprz1* | 0 | 0.3698997 | 0.192 | 0.043 | 5 |
| *Slc1a3* | 0 | 0.3642971 | 0.152 | 0.01 | 5 |
| *mt-Co1* | 0 | 0.3642065 | 0.42 | 0.417 | 5 |
| *Agt* | 0 | 0.362874 | 0.137 | 0.003 | 5 |
| *Gpm6b1* | 0 | 0.3461048 | 0.258 | 0.103 | 5 |
| *Slc6a1* | 0 | 0.3452291 | 0.178 | 0.053 | 5 |
| *Syne11* | 0 | 0.3415591 | 0.468 | 0.462 | 5 |
| *Acsl3* | 0 | 0.3359276 | 0.203 | 0.105 | 5 |
| *Lrp1* | 0 | 0.3327339 | 0.311 | 0.282 | 5 |
| *Slco1a4* | 0 | 0.3311611 | 0.121 | 0.003 | 5 |
| *Plpp3* | 0 | 0.3203941 | 0.14 | 0.009 | 5 |
| *Tril* | 0 | 0.3178307 | 0.144 | 0.01 | 5 |
| *Serpine2* | 0 | 0.314655 | 0.176 | 0.056 | 5 |
| *Epas1* | 0 | 0.3016294 | 0.138 | 0.02 | 5 |
| *Nnat* | 0 | 0.2996618 | 0.108 | 0.013 | 5 |
| *Rapgef3* | 0 | 0.2987869 | 0.139 | 0.014 | 5 |
| *Slc38a3* | 0 | 0.2909174 | 0.13 | 0.006 | 5 |
| *Utrn* | 0 | 0.2904571 | 0.183 | 0.077 | 5 |
| *Ptn1* | 0 | 0.2788613 | 0.188 | 0.103 | 5 |
| *Nwd1* | 0 | 0.2756162 | 0.139 | 0.049 | 5 |
| *Itm2a* | 0 | 0.2745194 | 0.106 | 0.01 | 5 |
| *mt-Nd5* | 0 | 0.2720926 | 0.393 | 0.406 | 5 |
| *Slc27a1* | 0 | 0.2688832 | 0.135 | 0.051 | 5 |
| *Sdc4* | 0 | 0.2600682 | 0.113 | 0.006 | 5 |
| *Slc2a1* | 0 | 0.2588932 | 0.116 | 0.015 | 5 |
| *Dbi* | 0 | 0.2564351 | 0.123 | 0.049 | 5 |
| *Trpm3* | 8.53350183497001e-320 | 0.3061331 | 0.192 | 0.073 | 5 |
| *Daam21* | 3.97E-303 | 0.2576742 | 0.14 | 0.045 | 5 |
| *Fn1* | 0 | 0.7072006 | 0.266 | 0.012 | 6 |
| *Sparcl13* | 0 | 0.6895945 | 0.797 | 0.45 | 6 |
| *Maf* | 0 | 0.5596038 | 0.514 | 0.051 | 6 |
| *Hcn1* | 0 | 0.5393843 | 0.518 | 0.134 | 6 |
| *Ndrg41* | 0 | 0.5139276 | 0.737 | 0.445 | 6 |
| *Nefh1* | 0 | 0.5119571 | 0.654 | 0.339 | 6 |
| *Nefm2* | 0 | 0.4980926 | 0.745 | 0.486 | 6 |
| *Lgi2* | 0 | 0.4844967 | 0.48 | 0.156 | 6 |
| *Ldhb2* | 0 | 0.4824331 | 0.651 | 0.33 | 6 |
| *Mafb* | 0 | 0.465526 | 0.401 | 0.014 | 6 |
| *Ckb2* | 0 | 0.4379359 | 0.713 | 0.437 | 6 |
| *Map1a2* | 0 | 0.4194278 | 0.711 | 0.455 | 6 |
| *Lpgat1* | 0 | 0.4122993 | 0.481 | 0.23 | 6 |
| *Hhip* | 0 | 0.4004197 | 0.216 | 0.009 | 6 |
| *Atp1b12* | 0 | 0.3793687 | 0.908 | 0.721 | 6 |
| *Spp1* | 0 | 0.3702855 | 0.351 | 0.073 | 6 |
| *Pcp4l1* | 0 | 0.3627434 | 0.436 | 0.194 | 6 |
| *Rap1gds1* | 0 | 0.3358917 | 0.323 | 0.112 | 6 |
| *Cd24a* | 0 | 0.3063732 | 0.247 | 0.028 | 6 |
| *Pvalb1* | 0 | 0.2977513 | 0.38 | 0.115 | 6 |
| *Sema7a* | 0 | 0.2931878 | 0.333 | 0.076 | 6 |
| *Lifr* | 0 | 0.2900475 | 0.338 | 0.089 | 6 |
| *Tcf4* | 0 | 0.2879863 | 0.429 | 0.154 | 6 |
| *Sphkap1* | 0 | 0.2814172 | 0.359 | 0.086 | 6 |
| *Rcan2* | 0 | 0.2679705 | 0.488 | 0.228 | 6 |
| *Gm205942* | 1.88E-254 | 0.3430948 | 0.789 | 0.618 | 6 |
| *Atp1a32* | 1.33E-253 | 0.3597125 | 0.721 | 0.506 | 6 |
| *mt-Nd11* | 4.26E-241 | 0.2693782 | 0.592 | 0.386 | 6 |
| *Nefl2* | 2.18E-227 | 0.3137003 | 0.573 | 0.374 | 6 |
| *Chgb1* | 0 | 0.7663805 | 0.7 | 0.3 | 7 |
| *Map1b1* | 0 | 0.5038042 | 0.855 | 0.706 | 7 |
| *Slc24a21* | 0 | 0.5022044 | 0.605 | 0.316 | 7 |
| *Snap252* | 0 | 0.4525786 | 0.806 | 0.627 | 7 |
| *Cplx21* | 0 | 0.4433372 | 0.47 | 0.131 | 7 |
| *Slc17a7* | 0 | 0.4349322 | 0.398 | 0.017 | 7 |
| *Pcp4* | 0 | 0.3590712 | 0.333 | 0.092 | 7 |
| *Gls* | 0 | 0.3527068 | 0.515 | 0.219 | 7 |
| *Sv2b* | 0 | 0.3437068 | 0.409 | 0.105 | 7 |
| *Spp11* | 0 | 0.3404474 | 0.342 | 0.074 | 7 |
| *Chd7* | 0 | 0.3316383 | 0.271 | 0.052 | 7 |
| *Syt1* | 0 | 0.3221465 | 0.462 | 0.178 | 7 |
| *Igfbp5* | 0 | 0.3151454 | 0.278 | 0.023 | 7 |
| *Zic1* | 0 | 0.2792836 | 0.206 | 0.016 | 7 |
| *Cpe* | 1.80E-266 | 0.2703337 | 0.543 | 0.315 | 7 |
| *Syne12* | 2.09E-265 | 0.3894454 | 0.669 | 0.456 | 7 |
| *Atp1b13* | 1.77E-261 | 0.3342662 | 0.864 | 0.723 | 7 |
| *Ndrg42* | 5.55E-236 | 0.3163572 | 0.644 | 0.448 | 7 |
| *Nefh2* | 1.86E-228 | 0.2723553 | 0.561 | 0.343 | 7 |
| *Dnm11* | 6.44E-223 | 0.3008523 | 0.552 | 0.347 | 7 |
| *Calm11* | 1.15E-216 | 0.3708707 | 0.794 | 0.667 | 7 |
| *Napb1* | 2.85E-207 | 0.2796105 | 0.631 | 0.447 | 7 |
| *Atp1a33* | 3.42E-125 | 0.2994903 | 0.661 | 0.509 | 7 |
| *Nefm3* | 0 | 1.7615498 | 0.976 | 0.485 | 8 |
| *Nefl3* | 0 | 1.680043 | 0.944 | 0.369 | 8 |
| *Map1b2* | 0 | 1.4496842 | 0.994 | 0.704 | 8 |
| *Nefh3* | 0 | 1.3304945 | 0.913 | 0.338 | 8 |
| *Slc5a7* | 0 | 0.9078166 | 0.65 | 0.011 | 8 |
| *Stmn2* | 0 | 0.9042521 | 0.808 | 0.399 | 8 |
| *Acly* | 0 | 0.8692583 | 0.702 | 0.146 | 8 |
| *Prune2* | 0 | 0.8525777 | 0.658 | 0.172 | 8 |
| *Calm12* | 0 | 0.8293291 | 0.946 | 0.665 | 8 |
| *S100b1* | 0 | 0.7708839 | 0.662 | 0.139 | 8 |
| *Sncg* | 0 | 0.7306761 | 0.582 | 0.061 | 8 |
| *Caln1* | 0 | 0.704289 | 0.649 | 0.18 | 8 |
| *Fgf1* | 0 | 0.6903476 | 0.619 | 0.087 | 8 |
| *Tppp31* | 0 | 0.6638242 | 0.554 | 0.088 | 8 |
| *Prph* | 0 | 0.6044724 | 0.522 | 0.007 | 8 |
| *Tubb3* | 0 | 0.5901334 | 0.642 | 0.245 | 8 |
| *Calm22* | 0 | 0.5212103 | 0.824 | 0.528 | 8 |
| *Pcp4l11* | 0 | 0.5067066 | 0.451 | 0.196 | 8 |
| *Vamp12* | 0 | 0.4938005 | 0.691 | 0.349 | 8 |
| *Atp1a1* | 0 | 0.4863912 | 0.422 | 0.067 | 8 |
| *mt-Nd12* | 0 | 0.4760841 | 0.697 | 0.387 | 8 |
| *Ahnak2* | 0 | 0.4678602 | 0.395 | 0.017 | 8 |
| *Ywhag1* | 0 | 0.4623602 | 0.693 | 0.374 | 8 |
| *Actg1* | 0 | 0.4526164 | 0.621 | 0.294 | 8 |
| *Epb41l3* | 0 | 0.4479075 | 0.651 | 0.376 | 8 |
| *Kif5c* | 0 | 0.447675 | 0.63 | 0.307 | 8 |
| *mt-Cytb2* | 0 | 0.4439124 | 0.764 | 0.456 | 8 |
| *Dffa* | 0 | 0.4280913 | 0.413 | 0.075 | 8 |
| *mt-Nd51* | 0 | 0.4186614 | 0.712 | 0.4 | 8 |
| *Rgs7bp1* | 0 | 0.4116456 | 0.514 | 0.2 | 8 |
| *Cplx12* | 0 | 0.4108131 | 0.737 | 0.41 | 8 |
| *Tns1* | 0 | 0.4064747 | 0.438 | 0.032 | 8 |
| *Fxyd7* | 0 | 0.4029779 | 0.326 | 0.021 | 8 |
| *Map2* | 0 | 0.4013043 | 0.633 | 0.336 | 8 |
| *Uchl1* | 0 | 0.3851554 | 0.546 | 0.253 | 8 |
| *Slc18a3* | 0 | 0.3789193 | 0.398 | 0.003 | 8 |
| *Sv2c1* | 0 | 0.3731882 | 0.504 | 0.211 | 8 |
| *Tuba1a1* | 0 | 0.3690301 | 0.575 | 0.261 | 8 |
| *Chodl* | 0 | 0.366018 | 0.296 | 0.012 | 8 |
| *Calca* | 0 | 0.3606921 | 0.244 | 0.004 | 8 |
| *Lgals1* | 0 | 0.3576738 | 0.297 | 0.011 | 8 |
| *Clu1* | 0 | 0.3558484 | 0.53 | 0.225 | 8 |
| *Slc7a14* | 0 | 0.3557184 | 0.459 | 0.125 | 8 |
| *Dkk3* | 0 | 0.3495818 | 0.366 | 0.053 | 8 |
| *Plec* | 0 | 0.348697 | 0.475 | 0.192 | 8 |
| *Fam135b* | 0 | 0.3239991 | 0.442 | 0.129 | 8 |
| *Ret* | 0 | 0.308943 | 0.366 | 0.046 | 8 |
| *Htra11* | 0 | 0.3034084 | 0.371 | 0.029 | 8 |
| *Fstl1* | 0 | 0.2988288 | 0.417 | 0.119 | 8 |
| *Rgs4* | 0 | 0.2960419 | 0.371 | 0.081 | 8 |
| *Capns1* | 0 | 0.2954707 | 0.395 | 0.118 | 8 |
| *Anxa6* | 0 | 0.2950323 | 0.412 | 0.111 | 8 |
| *Susd2* | 0 | 0.2771392 | 0.334 | 0.032 | 8 |
| *Fbxo2* | 0 | 0.2735682 | 0.363 | 0.108 | 8 |
| *Lifr1* | 0 | 0.2688005 | 0.359 | 0.091 | 8 |
| *Nfia* | 0 | 0.2673963 | 0.332 | 0.079 | 8 |
| *Stk32a* | 0 | 0.2630793 | 0.267 | 0.009 | 8 |
| *Kcnk9* | 0 | 0.2618339 | 0.409 | 0.129 | 8 |
| *Cyb5r3* | 0 | 0.2597222 | 0.327 | 0.046 | 8 |
| *Calcb* | 0 | 0.2563324 | 0.263 | 0.002 | 8 |
| *Endod1* | 0.00E+00 | 0.2885554 | 0.46 | 0.19 | 8 |
| *Rab6b* | 5.57E-307 | 0.3266993 | 0.532 | 0.248 | 8 |
| *Tuba1b* | 5.64E-302 | 0.3465961 | 0.576 | 0.295 | 8 |
| *Sptbn1* | 5.84E-296 | 0.3690503 | 0.662 | 0.395 | 8 |
| *mt-Co11* | 1.60E-295 | 0.3758191 | 0.707 | 0.412 | 8 |
| *Disp2* | 1.46E-285 | 0.4065246 | 0.573 | 0.323 | 8 |
| *Sorl1* | 2.44E-284 | 0.3597243 | 0.563 | 0.301 | 8 |
| *Hspa12a* | 1.09E-278 | 0.2626278 | 0.465 | 0.203 | 8 |
| *Klc1* | 7.90E-266 | 0.289986 | 0.532 | 0.263 | 8 |
| *mt-Nd6* | 2.09E-261 | 0.4164883 | 0.556 | 0.296 | 8 |
| *mt-Nd4* | 1.05E-258 | 0.3396292 | 0.524 | 0.261 | 8 |
| *Nars1* | 3.10E-256 | 0.3403952 | 0.616 | 0.349 | 8 |
| *mt-Nd21* | 5.33E-252 | 0.3968357 | 0.582 | 0.317 | 8 |
| *1110008P14Rik* | 1.02E-250 | 0.2538771 | 0.266 | 0.087 | 8 |
| *Hsp90aa11* | 5.63E-243 | 0.3770925 | 0.765 | 0.557 | 8 |
| *Map7d21* | 5.14E-231 | 0.3180183 | 0.623 | 0.362 | 8 |
| *Gm205943* | 8.43E-230 | 0.3632201 | 0.849 | 0.618 | 8 |
| *Kif21a* | 1.18E-229 | 0.3265865 | 0.641 | 0.403 | 8 |
| *Calm31* | 2.57E-227 | 0.3245775 | 0.625 | 0.37 | 8 |
| *Glra12* | 2.15E-217 | 0.2504803 | 0.528 | 0.279 | 8 |
| *Hsp90ab11* | 1.30E-192 | 0.2963025 | 0.801 | 0.615 | 8 |
| *Syt111* | 2.54E-185 | 0.3116977 | 0.759 | 0.553 | 8 |
| *Pkm1* | 1.46E-180 | 0.2980566 | 0.707 | 0.5 | 8 |
| *Pja2* | 1.51E-172 | 0.2607769 | 0.59 | 0.366 | 8 |
| *Stmn32* | 1.01E-169 | 0.3034142 | 0.623 | 0.399 | 8 |
| *Sptan1* | 1.01E-169 | 0.2879788 | 0.641 | 0.439 | 8 |
| *Ubb* | 3.79E-167 | 0.2748471 | 0.597 | 0.377 | 8 |
| *Kif5a* | 4.44E-167 | 0.2540981 | 0.617 | 0.396 | 8 |
| *Dync1h1* | 1.64E-140 | 0.2842837 | 0.633 | 0.444 | 8 |
| *Slc25a41* | 2.83E-138 | 0.2611627 | 0.726 | 0.524 | 8 |
| *Lars21* | 1.17E-80 | 0.3756277 | 0.948 | 0.86 | 8 |
| *Ttr* | 0 | 5.5248098 | 0.987 | 0.136 | 9 |
| *Enpp21* | 0 | 3.2458434 | 0.985 | 0.116 | 9 |
| *Trpm31* | 0 | 1.4798515 | 0.901 | 0.063 | 9 |
| *Apoe1* | 0 | 1.1665227 | 0.814 | 0.06 | 9 |
| *Arl6ip1* | 0 | 0.998859 | 0.823 | 0.342 | 9 |
| *Kl* | 0 | 0.9728218 | 0.7 | 0.017 | 9 |
| *mt-Nd52* | 0 | 0.8574512 | 0.865 | 0.398 | 9 |
| *Clu2* | 0 | 0.8504709 | 0.785 | 0.221 | 9 |
| *1500015O10Rik* | 0 | 0.8440248 | 0.621 | 0.001 | 9 |
| *Bsg1* | 0 | 0.8281743 | 0.813 | 0.307 | 9 |
| *Igfbp2* | 0 | 0.7614576 | 0.583 | 0.003 | 9 |
| *Prlr* | 0 | 0.7344926 | 0.574 | 0.034 | 9 |
| *Hspa5* | 0 | 0.7204849 | 0.817 | 0.365 | 9 |
| *mt-Nd61* | 0 | 0.6708455 | 0.722 | 0.293 | 9 |
| *Folr1* | 0 | 0.6515169 | 0.524 | 0.001 | 9 |
| *Clic6* | 0 | 0.6493874 | 0.496 | 0.002 | 9 |
| *Pbxip11* | 0 | 0.6342494 | 0.552 | 0.017 | 9 |
| *Cst31* | 0 | 0.5943275 | 0.595 | 0.154 | 9 |
| *Hsp90b1* | 0 | 0.5700656 | 0.72 | 0.34 | 9 |
| *Gm205944* | 0 | 0.5534777 | 0.909 | 0.618 | 9 |
| *Col9a3* | 0 | 0.5530513 | 0.451 | 0.005 | 9 |
| *F5* | 0 | 0.5422409 | 0.448 | 0.001 | 9 |
| *Kcnj13* | 0 | 0.5318642 | 0.444 | 0.001 | 9 |
| *Abhd2* | 0 | 0.5180168 | 0.367 | 0.032 | 9 |
| *Cd63* | 0 | 0.5174269 | 0.468 | 0.025 | 9 |
| *Calr* | 0 | 0.5089094 | 0.628 | 0.276 | 9 |
| *Slc4a2* | 0 | 0.5041416 | 0.495 | 0.071 | 9 |
| *Kcne2* | 0 | 0.499141 | 0.424 | 0 | 9 |
| *Sostdc1* | 0 | 0.4843245 | 0.4 | 0.001 | 9 |
| *Slc12a21* | 0 | 0.4646736 | 0.535 | 0.098 | 9 |
| *mt-Co12* | 0 | 0.4639086 | 0.739 | 0.411 | 9 |
| *Ptgds2* | 0 | 0.4631587 | 0.617 | 0.108 | 9 |
| *mt-Cytb3* | 0 | 0.4605701 | 0.783 | 0.456 | 9 |
| *Ace* | 0 | 0.4370415 | 0.303 | 0.021 | 9 |
| *Slc16a2* | 0 | 0.4338758 | 0.402 | 0.006 | 9 |
| *Car12* | 0 | 0.4242717 | 0.356 | 0.004 | 9 |
| *Slc4a10* | 0 | 0.4239848 | 0.445 | 0.117 | 9 |
| *Htr2c* | 0 | 0.4238085 | 0.431 | 0.086 | 9 |
| *Ctsd* | 0 | 0.4174925 | 0.462 | 0.117 | 9 |
| *Ezr* | 0 | 0.3879193 | 0.339 | 0.003 | 9 |
| *Lamb1* | 0 | 0.3734095 | 0.329 | 0.017 | 9 |
| *Cab39l* | 0 | 0.366835 | 0.374 | 0.048 | 9 |
| *Itpr1* | 0 | 0.3662083 | 0.366 | 0.067 | 9 |
| *Slc4a5* | 0 | 0.3541353 | 0.305 | 0.001 | 9 |
| *Col8a1* | 0 | 0.3474446 | 0.324 | 0.009 | 9 |
| *Cgnl1* | 0 | 0.3466997 | 0.325 | 0.083 | 9 |
| *Otx2* | 0 | 0.3452171 | 0.318 | 0.001 | 9 |
| *Slco1c1* | 0 | 0.3436034 | 0.312 | 0.005 | 9 |
| *Ifi27* | 0 | 0.3364907 | 0.333 | 0.032 | 9 |
| *Sparc1* | 0 | 0.3354434 | 0.378 | 0.039 | 9 |
| *Sod3* | 0 | 0.3312557 | 0.297 | 0.003 | 9 |
| *Wls* | 0 | 0.325979 | 0.326 | 0.022 | 9 |
| *Tbc1d9* | 0 | 0.3118417 | 0.311 | 0.068 | 9 |
| *Atp1a11* | 0 | 0.3046613 | 0.29 | 0.07 | 9 |
| *Rdh5* | 0 | 0.3028931 | 0.279 | 0.002 | 9 |
| *Slc13a4* | 0 | 0.3025639 | 0.282 | 0.013 | 9 |
| *Ccnd2* | 0 | 0.2963421 | 0.288 | 0.029 | 9 |
| *Ppp1r1b* | 0 | 0.2934323 | 0.262 | 0.005 | 9 |
| *Fxyd1* | 0 | 0.2914018 | 0.285 | 0.004 | 9 |
| *Dnajc3* | 0 | 0.2905242 | 0.35 | 0.091 | 9 |
| *Msx1* | 0 | 0.2867925 | 0.264 | 0.001 | 9 |
| *Cdkn1c* | 0 | 0.2862008 | 0.258 | 0.004 | 9 |
| *Slc31a1* | 0 | 0.2846267 | 0.271 | 0.016 | 9 |
| *Car21* | 0 | 0.279256 | 0.433 | 0.077 | 9 |
| *Mfrp* | 0 | 0.2777927 | 0.178 | 0.002 | 9 |
| *Sulf1* | 0 | 0.274706 | 0.243 | 0.006 | 9 |
| *Abca4* | 0 | 0.2708458 | 0.225 | 0.003 | 9 |
| *Stk39* | 0 | 0.2640549 | 0.306 | 0.088 | 9 |
| *Strip2* | 0 | 0.2632678 | 0.272 | 0.059 | 9 |
| *Syne2* | 0 | 0.2618109 | 0.258 | 0.019 | 9 |
| *Plxnb2* | 0 | 0.2608731 | 0.238 | 0.029 | 9 |
| *Col8a2* | 0 | 0.2550985 | 0.229 | 0.002 | 9 |
| *Cd59a* | 1.55595662130222e-314 | 0.2833584 | 0.392 | 0.136 | 9 |
| *mt-Nd41* | 4.38E-294 | 0.3260103 | 0.547 | 0.261 | 9 |
| *mt-Nd22* | 9.43E-292 | 0.3695885 | 0.61 | 0.317 | 9 |
| *Tmem721* | 6.24E-286 | 0.3403095 | 0.588 | 0.297 | 9 |
| *Zbtb201* | 2.44E-268 | 0.3166631 | 0.643 | 0.35 | 9 |
| *Eef1a11* | 5.14E-265 | 0.2800504 | 0.454 | 0.19 | 9 |
| *Psap* | 1.67E-257 | 0.3603301 | 0.621 | 0.358 | 9 |
| *Ahcyl2* | 3.56E-254 | 0.2552917 | 0.315 | 0.111 | 9 |
| *Slc22a17* | 6.91E-246 | 0.3033416 | 0.538 | 0.283 | 9 |
| *Atp2b3* | 4.32E-244 | 0.298423 | 0.34 | 0.152 | 9 |
| *Lars22* | 2.06E-80 | 0.4367687 | 0.956 | 0.86 | 9 |
| *Nrcam* | 0 | 1.2774311 | 0.788 | 0.224 | 10 |
| *Scn3a* | 0 | 1.101072 | 0.745 | 0.08 | 10 |
| *Slc1a21* | 0 | 0.730126 | 0.545 | 0.054 | 10 |
| *Rph3a1* | 0 | 0.6743113 | 0.589 | 0.239 | 10 |
| *Cacna1e* | 0 | 0.6706936 | 0.575 | 0.143 | 10 |
| *Calb1* | 0 | 0.6062498 | 0.466 | 0.031 | 10 |
| *Arpp211* | 0 | 0.5819031 | 0.547 | 0.145 | 10 |
| *Syt12* | 0 | 0.5563635 | 0.526 | 0.18 | 10 |
| *Crh* | 0 | 0.528979 | 0.354 | 0.009 | 10 |
| *Nell2* | 0 | 0.5176584 | 0.398 | 0.056 | 10 |
| *Msi2* | 0 | 0.5121358 | 0.514 | 0.189 | 10 |
| *Foxp2* | 0 | 0.5040948 | 0.425 | 0.029 | 10 |
| *Gpr88* | 0 | 0.4977039 | 0.372 | 0.002 | 10 |
| *Cplx22* | 0 | 0.4607197 | 0.449 | 0.135 | 10 |
| *Gpr26* | 0 | 0.4283786 | 0.347 | 0.027 | 10 |
| *Pex5l1* | 0 | 0.4144912 | 0.422 | 0.103 | 10 |
| *Htr5b* | 0 | 0.4015871 | 0.326 | 0.001 | 10 |
| *Plcxd2* | 0 | 0.3858854 | 0.362 | 0.089 | 10 |
| *Kit* | 0 | 0.3382917 | 0.293 | 0.016 | 10 |
| *Gabrg1* | 0 | 0.3347289 | 0.304 | 0.024 | 10 |
| *Pou4f1* | 0 | 0.3278475 | 0.292 | 0.006 | 10 |
| *Foxp1* | 0 | 0.3243898 | 0.308 | 0.054 | 10 |
| *Cacna1g* | 0 | 0.2996582 | 0.276 | 0.045 | 10 |
| *Kcng1* | 0 | 0.2746611 | 0.257 | 0.008 | 10 |
| *Cnih3* | 0 | 0.2632164 | 0.258 | 0.011 | 10 |
| *Ajap1* | 0 | 0.2603678 | 0.269 | 0.042 | 10 |
| *Lmo7* | 0 | 0.2591114 | 0.203 | 0.008 | 10 |
| *Dnah11* | 0 | 0.2583439 | 0.19 | 0.002 | 10 |
| *C1ql1* | 0 | 0.2538234 | 0.244 | 0.004 | 10 |
| *Smpd3* | 0 | 0.251076 | 0.26 | 0.038 | 10 |
| *Calb2* | 1.68970450877706e-321 | 0.3260941 | 0.349 | 0.081 | 10 |
| *Nav3* | 2.41207098104162e-314 | 0.3245279 | 0.334 | 0.079 | 10 |
| *Adgra1* | 2.52E-268 | 0.2809944 | 0.306 | 0.081 | 10 |
| *Kcnh7* | 5.64E-253 | 0.2745858 | 0.3 | 0.077 | 10 |
| *Zfp462* | 8.68E-233 | 0.2779432 | 0.313 | 0.091 | 10 |
| *Ptprd1* | 8.32E-229 | 0.4075489 | 0.598 | 0.315 | 10 |
| *Cpne6* | 6.30E-221 | 0.2523094 | 0.269 | 0.068 | 10 |
| *Fbxw7* | 4.04E-214 | 0.2960523 | 0.364 | 0.143 | 10 |
| *Pcp4l12* | 1.81E-213 | 0.3971873 | 0.41 | 0.198 | 10 |
| *Snhg111* | 5.51E-206 | 0.496118 | 0.978 | 0.863 | 10 |
| *Kmt2c* | 1.72E-190 | 0.3067879 | 0.396 | 0.161 | 10 |
| *Gpm6a* | 5.23E-187 | 0.2938516 | 0.395 | 0.164 | 10 |
| *Tsc22d1* | 2.12E-182 | 0.2741192 | 0.358 | 0.157 | 10 |
| *Lgi21* | 6.29E-168 | 0.2698586 | 0.357 | 0.163 | 10 |
| *Cntnap2* | 1.25E-139 | 0.2832811 | 0.435 | 0.251 | 10 |
| *Akap6* | 1.42E-131 | 0.2875968 | 0.414 | 0.24 | 10 |
| *Ntrk3* | 1.88E-125 | 0.2500144 | 0.399 | 0.204 | 10 |
| *Rab3c* | 5.80E-123 | 0.2532833 | 0.422 | 0.231 | 10 |
| *Zbtb202* | 1.67E-73 | 0.2667237 | 0.503 | 0.353 | 10 |
| *Nat8l1* | 0 | 1.7055516 | 0.764 | 0.277 | 11 |
| *Stx1b* | 0 | 1.0309443 | 0.694 | 0.299 | 11 |
| *Lars23* | 0 | 0.9530447 | 0.951 | 0.86 | 11 |
| *Vmn1r206* | 0 | 0.8928626 | 0.557 | 0.024 | 11 |
| *Spock2* | 0 | 0.797972 | 0.691 | 0.39 | 11 |
| *Fyco1* | 0 | 0.7464235 | 0.556 | 0.129 | 11 |
| *Dcbld2* | 0 | 0.6233987 | 0.43 | 0.052 | 11 |
| *Mtch1* | 0 | 0.6186533 | 0.497 | 0.223 | 11 |
| *Spop* | 0 | 0.5920649 | 0.5 | 0.191 | 11 |
| *Dynll2* | 0 | 0.579814 | 0.543 | 0.339 | 11 |
| *Srgap3* | 0 | 0.5705221 | 0.511 | 0.219 | 11 |
| *Ctnnd2* | 0 | 0.488252 | 0.413 | 0.129 | 11 |
| *Cd22* | 0 | 0.4447912 | 0.27 | 0.01 | 11 |
| *Tgfbi* | 0 | 0.3447108 | 0.229 | 0.025 | 11 |
| *Abcc4* | 0 | 0.3030108 | 0.197 | 0.012 | 11 |
| *Rgs51* | 0 | 0.2544776 | 0.191 | 0.011 | 11 |
| *Mdga2* | 7.34132143155508e-319 | 0.4749736 | 0.429 | 0.165 | 11 |
| *Usp53* | 1.51E-303 | 0.3861844 | 0.298 | 0.065 | 11 |
| *Shq1* | 5.82E-281 | 0.3338673 | 0.241 | 0.045 | 11 |
| *Lonrf1* | 1.02E-274 | 0.3610977 | 0.287 | 0.072 | 11 |
| *Fam219a* | 1.57E-273 | 0.357584 | 0.324 | 0.111 | 11 |
| *Slc16a10* | 9.07E-271 | 0.3423832 | 0.247 | 0.05 | 11 |
| *Shld1* | 1.83E-248 | 0.2934787 | 0.204 | 0.035 | 11 |
| *Tmem132d* | 1.55E-246 | 0.281562 | 0.199 | 0.03 | 11 |
| *Arf3* | 1.18E-244 | 0.3719308 | 0.389 | 0.182 | 11 |
| *Lhfpl4* | 7.28E-242 | 0.3507863 | 0.32 | 0.117 | 11 |
| *Slc9b2* | 4.73E-232 | 0.26379 | 0.192 | 0.029 | 11 |
| *Mrgpre* | 1.63E-229 | 0.264325 | 0.197 | 0.034 | 11 |
| *Pik3c2a* | 3.39E-210 | 0.3078433 | 0.265 | 0.072 | 11 |
| *Madd* | 2.06E-202 | 0.3710589 | 0.391 | 0.204 | 11 |
| *Fam160b2* | 3.69E-199 | 0.3052518 | 0.265 | 0.082 | 11 |
| *Mroh1* | 2.08E-188 | 0.3731423 | 0.375 | 0.183 | 11 |
| *Dzank1* | 2.94E-147 | 0.3014214 | 0.406 | 0.259 | 11 |
| *Celf41* | 6.04E-135 | 0.3726442 | 0.456 | 0.3 | 11 |
| *Gdi1* | 4.88E-116 | 0.2742928 | 0.465 | 0.343 | 11 |
| *Atp1a34* | 3.48E-99 | 0.3178588 | 0.663 | 0.511 | 11 |
| *Pkd1* | 1.79E-89 | 0.2681207 | 0.362 | 0.205 | 11 |
| *Slc6a51* | 0 | 1.0958838 | 0.891 | 0.156 | 12 |
| *Sparcl14* | 0 | 1.0323294 | 0.97 | 0.455 | 12 |
| *Spp12* | 0 | 0.9663713 | 0.864 | 0.074 | 12 |
| *Ndrg43* | 0 | 0.8950686 | 0.942 | 0.449 | 12 |
| *Map1a3* | 0 | 0.8822419 | 0.947 | 0.458 | 12 |
| *Nefh4* | 0 | 0.8305947 | 0.928 | 0.343 | 12 |
| *Ckb3* | 0 | 0.8289576 | 0.939 | 0.441 | 12 |
| *Calb11* | 0 | 0.7896467 | 0.714 | 0.031 | 12 |
| *Lamp51* | 0 | 0.7547427 | 0.878 | 0.216 | 12 |
| *Gm205945* | 0 | 0.6930772 | 0.954 | 0.62 | 12 |
| *Atp1b14* | 0 | 0.6395438 | 0.99 | 0.725 | 12 |
| *mt-Cytb4* | 0 | 0.6235564 | 0.925 | 0.458 | 12 |
| *Nefm4* | 0 | 0.6133711 | 0.947 | 0.49 | 12 |
| *Ldhb3* | 0 | 0.6117885 | 0.872 | 0.335 | 12 |
| *mt-Co13* | 0 | 0.6095623 | 0.901 | 0.413 | 12 |
| *mt-Nd13* | 0 | 0.6000307 | 0.862 | 0.389 | 12 |
| *Cd24a1* | 0 | 0.5902107 | 0.63 | 0.029 | 12 |
| *mt-Nd53* | 0 | 0.5291358 | 0.872 | 0.402 | 12 |
| *Pcp4l13* | 0 | 0.517397 | 0.702 | 0.197 | 12 |
| *Cplx13* | 0 | 0.5102504 | 0.908 | 0.411 | 12 |
| *Doc2g* | 0 | 0.5027164 | 0.436 | 0.007 | 12 |
| *Kcnc31* | 0 | 0.4906735 | 0.805 | 0.276 | 12 |
| *Ttn* | 0 | 0.4816575 | 0.447 | 0.025 | 12 |
| *Sv2c2* | 0 | 0.4757905 | 0.744 | 0.212 | 12 |
| *Pvalb2* | 0 | 0.423102 | 0.605 | 0.118 | 12 |
| *Fgf11* | 0 | 0.4163892 | 0.618 | 0.092 | 12 |
| *Kcna11* | 0 | 0.4152854 | 0.804 | 0.295 | 12 |
| *Ina2* | 0 | 0.4123801 | 0.75 | 0.278 | 12 |
| *Vamp13* | 0 | 0.4072803 | 0.847 | 0.351 | 12 |
| *Rcan21* | 0 | 0.4012657 | 0.736 | 0.231 | 12 |
| *Nrxn3* | 0 | 0.3821606 | 0.872 | 0.376 | 12 |
| *Dner* | 0 | 0.3754797 | 0.638 | 0.129 | 12 |
| *Lpgat11* | 0 | 0.3600748 | 0.691 | 0.234 | 12 |
| *Lgi22* | 0 | 0.3555206 | 0.638 | 0.162 | 12 |
| *Ghitm1* | 0 | 0.3490615 | 0.812 | 0.345 | 12 |
| *Syt21* | 0 | 0.3391313 | 0.82 | 0.332 | 12 |
| *Tenm4* | 0 | 0.333517 | 0.638 | 0.194 | 12 |
| *Pacsin2* | 0 | 0.3125246 | 0.59 | 0.113 | 12 |
| *Elovl5* | 0 | 0.3066045 | 0.52 | 0.07 | 12 |
| *Rgs7bp2* | 0 | 0.2964391 | 0.644 | 0.202 | 12 |
| *Hspg2* | 0 | 0.2742651 | 0.314 | 0.005 | 12 |
| *Ttll7* | 0 | 0.2647731 | 0.674 | 0.235 | 12 |
| *Mdh11* | 2.27270197086973e-322 | 0.4502565 | 0.846 | 0.394 | 12 |
| *Scn8a1* | 3.4090529563046e-322 | 0.2820236 | 0.788 | 0.343 | 12 |
| *mt-Nd42* | 9.78249978765668e-322 | 0.3718868 | 0.713 | 0.262 | 12 |
| *Atp2a21* | 1.82557256138341e-320 | 0.3863988 | 0.829 | 0.372 | 12 |
| *Scn4b1* | 4.67703923514476e-315 | 0.2729264 | 0.532 | 0.143 | 12 |
| *Atp1a35* | 6.29094088921395e-314 | 0.7133905 | 0.916 | 0.509 | 12 |
| *Psap1* | 1.12E-307 | 0.2925393 | 0.797 | 0.359 | 12 |
| *Clstn11* | 5.22E-305 | 0.3281631 | 0.806 | 0.364 | 12 |
| *Cend1* | 5.78E-305 | 0.2533888 | 0.665 | 0.244 | 12 |
| *Pkm2* | 4.74E-304 | 0.4148134 | 0.919 | 0.5 | 12 |
| *Atp5b2* | 6.34E-300 | 0.4577356 | 0.9 | 0.493 | 12 |
| *Myo5a* | 2.58E-298 | 0.2777 | 0.73 | 0.299 | 12 |
| *mt-Nd23* | 1.55E-297 | 0.5236348 | 0.75 | 0.319 | 12 |
| *Ywhag2* | 1.97E-292 | 0.2905228 | 0.805 | 0.376 | 12 |
| *Map1b3* | 1.72E-290 | 0.445745 | 0.982 | 0.707 | 12 |
| *Ctsb* | 2.54E-289 | 0.2507843 | 0.811 | 0.393 | 12 |
| *Glra13* | 4.36E-281 | 0.3094168 | 0.701 | 0.28 | 12 |
| *Aplp2* | 2.13E-271 | 0.2744142 | 0.828 | 0.42 | 12 |
| *Kcnc1* | 2.02E-268 | 0.3417431 | 0.754 | 0.335 | 12 |
| *Nars2* | 3.36E-263 | 0.2620816 | 0.753 | 0.351 | 12 |
| *Dnm12* | 5.51E-262 | 0.2891962 | 0.75 | 0.349 | 12 |
| *Snap253* | 2.92E-260 | 0.398959 | 0.968 | 0.629 | 12 |
| *Map7d22* | 2.46E-258 | 0.2653492 | 0.764 | 0.364 | 12 |
| *mt-Nd62* | 1.05E-251 | 0.4035462 | 0.701 | 0.297 | 12 |
| *Spock21* | 9.19E-251 | 0.337828 | 0.791 | 0.39 | 12 |
| *Slc25a42* | 8.21E-242 | 0.4874889 | 0.89 | 0.525 | 12 |
| *Hspa81* | 3.03E-235 | 0.4084137 | 0.837 | 0.452 | 12 |
| *Stmn33* | 1.58E-198 | 0.2987345 | 0.759 | 0.4 | 12 |
| *Dst* | 1.34E-193 | 0.2957473 | 0.888 | 0.553 | 12 |
| *Hsp90ab12* | 3.86E-187 | 0.2540817 | 0.926 | 0.616 | 12 |
| *Calm13* | 7.61E-138 | 0.2601454 | 0.925 | 0.668 | 12 |
| *Lars24* | 2.81E-90 | 0.3582159 | 0.991 | 0.86 | 12 |
| *Gapdh1* | 4.79E-87 | 0.3209065 | 0.657 | 0.415 | 12 |
| *Atp2b1* | 0 | 1.1125109 | 0.625 | 0.332 | 13 |
| *Grid2* | 0 | 1.0873093 | 0.651 | 0.074 | 13 |
| *Prkcd* | 0 | 0.8157546 | 0.392 | 0.01 | 13 |
| *Anks1b* | 0 | 0.7601955 | 0.502 | 0.111 | 13 |
| *Cdh6* | 0 | 0.7054873 | 0.442 | 0.042 | 13 |
| *Bod1l* | 0 | 0.7023377 | 0.473 | 0.203 | 13 |
| *Car8* | 0 | 0.6920567 | 0.313 | 0.015 | 13 |
| *Dgkh* | 0 | 0.6657198 | 0.447 | 0.054 | 13 |
| *Ryr1* | 0 | 0.6467085 | 0.304 | 0.007 | 13 |
| *Adarb2* | 0 | 0.5900202 | 0.381 | 0.051 | 13 |
| *Gria21* | 2.31173315689119e-319 | 0.7093644 | 0.654 | 0.418 | 13 |
| *Itpr11* | 9.17E-304 | 0.5869735 | 0.341 | 0.07 | 13 |
| *Grm1* | 3.61E-232 | 0.465593 | 0.358 | 0.128 | 13 |
| *Tnr* | 3.05E-228 | 0.552817 | 0.388 | 0.127 | 13 |
| *Ogfrl1* | 8.27E-190 | 0.408877 | 0.286 | 0.087 | 13 |
| *Cttnbp2* | 3.02E-181 | 0.4664438 | 0.376 | 0.118 | 13 |
| *Trim9* | 8.27E-173 | 0.4336005 | 0.343 | 0.124 | 13 |
| *Sptbn2* | 2.22E-171 | 0.4270163 | 0.328 | 0.135 | 13 |
| *Map4* | 4.48E-164 | 0.4223111 | 0.389 | 0.218 | 13 |
| *Kalrn* | 6.16E-147 | 0.4103535 | 0.361 | 0.127 | 13 |
| *Vav3* | 8.41E-141 | 0.2847696 | 0.196 | 0.033 | 13 |
| *Shank2* | 8.55E-141 | 0.3175443 | 0.227 | 0.045 | 13 |
| *Rora* | 7.23E-140 | 0.3847959 | 0.317 | 0.126 | 13 |
| *Snhg112* | 8.93E-140 | 0.5868579 | 0.973 | 0.863 | 13 |
| *Pcp41* | 1.67E-131 | 0.4350893 | 0.254 | 0.097 | 13 |
| *Thsd7a* | 1.75E-129 | 0.3622019 | 0.315 | 0.129 | 13 |
| *Rreb1* | 5.88E-129 | 0.2867385 | 0.178 | 0.025 | 13 |
| *Grin2b* | 5.23E-124 | 0.36892 | 0.32 | 0.149 | 13 |
| *Rasa4* | 8.78E-103 | 0.2659244 | 0.194 | 0.041 | 13 |
| *Tshz2* | 1.56E-99 | 0.3142172 | 0.249 | 0.099 | 13 |
| *Sorl11* | 1.05E-95 | 0.3516879 | 0.445 | 0.305 | 13 |
| *Inpp5j* | 1.31E-94 | 0.2978166 | 0.244 | 0.088 | 13 |
| *Ywhah* | 4.16E-94 | 0.2638047 | 0.327 | 0.289 | 13 |
| *Exoc6b* | 5.67E-94 | 0.302147 | 0.26 | 0.114 | 13 |
| *Sipa1l3* | 1.03E-88 | 0.2577282 | 0.22 | 0.066 | 13 |
| *Frmd4a* | 1.33E-85 | 0.3317603 | 0.307 | 0.144 | 13 |
| *Cdc42bpa* | 7.50E-85 | 0.3280913 | 0.363 | 0.204 | 13 |
| *Nsg1* | 2.60E-83 | 0.2624021 | 0.323 | 0.236 | 13 |
| *Auts2* | 2.50E-79 | 0.3059808 | 0.284 | 0.128 | 13 |
| *Kcnma1* | 1.95E-74 | 0.2800633 | 0.303 | 0.157 | 13 |
| *Cpe1* | 5.51E-68 | 0.2531741 | 0.381 | 0.321 | 13 |
| *Shank1* | 1.09E-65 | 0.3032743 | 0.388 | 0.269 | 13 |
| *Arhgef12* | 2.15E-60 | 0.258207 | 0.299 | 0.213 | 13 |
| *Unc80* | 4.36E-57 | 0.2639798 | 0.442 | 0.349 | 13 |
| *Ccdc88a* | 9.44E-56 | 0.2786791 | 0.314 | 0.199 | 13 |
| *Ndrg44* | 2.94E-49 | 0.260818 | 0.516 | 0.453 | 13 |
| *Tnr1* | 0 | 1.3532412 | 0.605 | 0.125 | 14 |
| *Nfasc1* | 0 | 1.2924187 | 0.454 | 0.267 | 14 |
| *Gjc31* | 0 | 1.0037385 | 0.424 | 0.046 | 14 |
| *Vcan* | 0 | 0.9512637 | 0.39 | 0.017 | 14 |
| *Frmd4a1* | 0 | 0.9455795 | 0.38 | 0.144 | 14 |
| *Sirt21* | 0 | 0.912696 | 0.386 | 0.08 | 14 |
| *Ptprz11* | 0 | 0.8346418 | 0.349 | 0.046 | 14 |
| *Gpr17* | 0 | 0.8178256 | 0.272 | 0.002 | 14 |
| *Marcks* | 0 | 0.7985201 | 0.325 | 0.045 | 14 |
| *Bcas1* | 0 | 0.7610374 | 0.284 | 0.016 | 14 |
| *Pdgfra* | 0 | 0.7543389 | 0.215 | 0.005 | 14 |
| *Ppfibp1* | 0 | 0.6943152 | 0.309 | 0.019 | 14 |
| *Fyn* | 0 | 0.6609994 | 0.283 | 0.053 | 14 |
| *Tns3* | 0 | 0.6198661 | 0.267 | 0.014 | 14 |
| *Itpr2* | 0 | 0.5977648 | 0.265 | 0.03 | 14 |
| *Cspg4* | 0 | 0.5137608 | 0.172 | 0.003 | 14 |
| *Bcan1* | 1.97626258336499e-323 | 0.7251053 | 0.354 | 0.125 | 14 |
| *Kank1* | 1.90E-293 | 0.4255857 | 0.187 | 0.009 | 14 |
| *Qk2* | 9.25E-287 | 0.8312374 | 0.447 | 0.102 | 14 |
| *Mbp2* | 2.92E-278 | 1.0087408 | 0.477 | 0.186 | 14 |
| *Enpp6* | 1.38E-270 | 0.5392537 | 0.217 | 0.013 | 14 |
| *Neu4* | 2.51E-259 | 0.3235842 | 0.12 | 0.001 | 14 |
| *Cd812* | 4.36E-259 | 0.6383522 | 0.38 | 0.183 | 14 |
| *Lrp11* | 1.81E-244 | 0.5703849 | 0.29 | 0.283 | 14 |
| *Tcf7l2* | 1.84E-238 | 0.3672304 | 0.155 | 0.006 | 14 |
| *Gpr37l11* | 1.03E-235 | 0.4903116 | 0.221 | 0.015 | 14 |
| *Plp11* | 2.14E-211 | 0.9779335 | 0.606 | 0.268 | 14 |
| *Plxnb31* | 1.15E-209 | 0.5915398 | 0.22 | 0.021 | 14 |
| *Mical3* | 2.62E-203 | 0.4841772 | 0.276 | 0.146 | 14 |
| *Cnp1* | 5.46E-203 | 0.8255272 | 0.363 | 0.081 | 14 |
| *Slc44a11* | 5.84E-199 | 0.48689 | 0.229 | 0.05 | 14 |
| *Cntn1* | 7.03E-194 | 0.3477394 | 0.316 | 0.286 | 14 |
| *Serpine21* | 1.04E-189 | 0.463918 | 0.22 | 0.059 | 14 |
| *Mpzl1* | 1.41E-189 | 0.3449105 | 0.15 | 0.01 | 14 |
| *Olig11* | 4.60E-189 | 0.4088429 | 0.187 | 0.014 | 14 |
| *Epn2* | 9.90E-189 | 0.4566033 | 0.219 | 0.044 | 14 |
| *Cemip2* | 2.33E-185 | 0.3978554 | 0.134 | 0.03 | 14 |
| *Sema4d* | 1.45E-180 | 0.41474 | 0.179 | 0.036 | 14 |
| *Sulf2* | 4.26E-180 | 0.3866972 | 0.185 | 0.042 | 14 |
| *Dock91* | 4.39E-177 | 0.510572 | 0.319 | 0.15 | 14 |
| *Cacng4* | 5.23E-176 | 0.3660899 | 0.146 | 0.022 | 14 |
| *Col9a31* | 1.29E-170 | 0.3352737 | 0.147 | 0.012 | 14 |
| *Epb41l2* | 1.18E-160 | 0.3857107 | 0.159 | 0.033 | 14 |
| *Tuba1a2* | 1.85E-158 | 0.366908 | 0.283 | 0.267 | 14 |
| *Xylt1* | 4.04E-158 | 0.3290188 | 0.132 | 0.024 | 14 |
| *Pik3r3* | 1.40E-156 | 0.3150863 | 0.127 | 0.039 | 14 |
| *Nav1* | 1.36E-155 | 0.3990551 | 0.293 | 0.201 | 14 |
| *Acsl31* | 1.17E-154 | 0.3782013 | 0.203 | 0.107 | 14 |
| *Trio* | 9.01E-153 | 0.3681115 | 0.271 | 0.19 | 14 |
| *Cd9* | 1.13E-148 | 0.2927605 | 0.117 | 0.009 | 14 |
| *Pcdh17* | 8.08E-146 | 0.3445803 | 0.202 | 0.141 | 14 |
| *Sema5a* | 1.58E-144 | 0.3585588 | 0.206 | 0.089 | 14 |
| *Ankrd281* | 1.10E-143 | 0.3999173 | 0.217 | 0.074 | 14 |
| *Olig2* | 1.82E-141 | 0.2793114 | 0.115 | 0.006 | 14 |
| *Mag1* | 7.97E-140 | 0.842908 | 0.325 | 0.09 | 14 |
| *Hip1r* | 1.93E-138 | 0.3327983 | 0.168 | 0.063 | 14 |
| *Sema6a* | 1.83E-137 | 0.3602354 | 0.171 | 0.049 | 14 |
| *Map4k4* | 1.36E-136 | 0.3920935 | 0.228 | 0.085 | 14 |
| *Son1* | 1.45E-134 | 0.332694 | 0.365 | 0.341 | 14 |
| *Arhgap31* | 4.74E-134 | 0.3162263 | 0.146 | 0.037 | 14 |
| *Col11a2* | 5.33E-134 | 0.3590702 | 0.139 | 0.017 | 14 |
| *Zeb21* | 5.40E-131 | 0.4512218 | 0.258 | 0.069 | 14 |
| *Rictor* | 1.18E-130 | 0.3471 | 0.194 | 0.09 | 14 |
| *Dock4* | 6.96E-129 | 0.3447094 | 0.194 | 0.092 | 14 |
| *Shisal1* | 7.16E-129 | 0.2685024 | 0.133 | 0.029 | 14 |
| *Ddx5* | 5.10E-126 | 0.3043107 | 0.369 | 0.365 | 14 |
| *Spon1* | 8.68E-120 | 0.2981022 | 0.145 | 0.043 | 14 |
| *Adamts41* | 1.20E-119 | 0.3744926 | 0.165 | 0.025 | 14 |
| *Hnrnpa2b1* | 2.40E-118 | 0.2546692 | 0.285 | 0.308 | 14 |
| *Ctsl* | 1.73E-116 | 0.2567533 | 0.146 | 0.082 | 14 |
| *Pcdh15* | 4.87E-116 | 0.2923265 | 0.137 | 0.059 | 14 |
| *Lcorl* | 6.19E-116 | 0.2605945 | 0.131 | 0.024 | 14 |
| *Fryl* | 3.37E-115 | 0.3081083 | 0.219 | 0.154 | 14 |
| *Nckap5* | 1.19E-114 | 0.2552841 | 0.13 | 0.025 | 14 |
| *Actb1* | 3.81E-109 | 0.3534303 | 0.524 | 0.528 | 14 |
| *Phldb11* | 2.18E-108 | 0.3369088 | 0.176 | 0.03 | 14 |
| *Chn2* | 1.57E-105 | 0.2714967 | 0.12 | 0.024 | 14 |
| *Man2a2* | 2.15E-104 | 0.285966 | 0.187 | 0.127 | 14 |
| *Sep-71* | 1.19E-102 | 0.2532509 | 0.287 | 0.292 | 14 |
| *Ncor2* | 2.04E-97 | 0.2801186 | 0.18 | 0.115 | 14 |
| *Ncam1* | 3.84E-96 | 0.2623187 | 0.334 | 0.331 | 14 |
| *Cldn111* | 1.45E-87 | 0.3507074 | 0.161 | 0.029 | 14 |
| *Kndc11* | 1.86E-84 | 0.2644373 | 0.19 | 0.174 | 14 |
| *Ugt8a1* | 1.01E-83 | 0.359458 | 0.206 | 0.066 | 14 |
| *Zbtb203* | 2.13E-82 | 0.253466 | 0.341 | 0.355 | 14 |
| *Utp14b* | 4.49E-82 | 0.2556812 | 0.131 | 0.048 | 14 |
| *Secisbp2l1* | 7.37E-82 | 0.2839502 | 0.153 | 0.071 | 14 |
| *Scd22* | 1.10E-73 | 0.3693815 | 0.382 | 0.262 | 14 |
| *Myrf1* | 1.12E-73 | 0.2602219 | 0.139 | 0.033 | 14 |
| *Tmeff21* | 1.98E-71 | 0.2624986 | 0.172 | 0.085 | 14 |
| *AC149090.11* | 2.41E-70 | 0.2727262 | 0.331 | 0.34 | 14 |
| *S100b2* | 6.01E-68 | 0.3718325 | 0.253 | 0.147 | 14 |
| *Aatk1* | 1.27E-65 | 0.2534311 | 0.175 | 0.117 | 14 |
| *Ptma1* | 1.10E-62 | 0.2533219 | 0.202 | 0.149 | 14 |
| *Sparc2* | 1.64E-57 | 0.3038959 | 0.15 | 0.044 | 14 |
| *Slc12a22* | 3.45E-47 | 0.277781 | 0.199 | 0.105 | 14 |
| *Mobp2* | 3.04E-30 | 0.3282238 | 0.22 | 0.132 | 14 |
